# Predictors of validity and reliability of a physical activity record in adolescents

**DOI:** 10.1186/1471-2458-13-1109

**Published:** 2013-12-01

**Authors:** Roosmarijn Verstraeten, Carl Lachat, Angélica Ochoa-Avilés, Maria Hagströmer, Lieven Huybregts, Susana Andrade, Silvana Donoso, John Van Camp, Lea Maes, Patrick Kolsteren

**Affiliations:** 1 Nutrition and Child Health Unit, Department of Public Health, Institute of Tropical Medicine, Nationalestraat 155, 2000, Antwerp, Belgium; 2 Department of Food Safety and Food Quality, Ghent University, Coupure Links 653, 9000 Ghent, Belgium; 3 Food, Nutrition and Health program, Universidad de Cuenca, Avenida 12 de Abril s/n Ciudadela Universitaria, Cuenca, Ecuador; 4 Department of Neurobiology, Care Sciences and Society, Division of Physiotherapy, Karolinska Institute, 23100, 141 83 Huddinge, Sweden; 5 Department of Public Health, Ghent University, De Pintelaan 185, 9000 Ghent, Belgium

**Keywords:** Accelerometers, Convenience, Diary, Ecuador, Low- and middle-income countries, Validity

## Abstract

**Background:**

Poor to moderate validity of self-reported physical activity instruments is commonly observed in young people in low- and middle-income countries. However, the reasons for such low validity have not been examined in detail. We tested the validity of a self-administered daily physical activity record in adolescents and assessed if personal characteristics or the convenience level of reporting physical activity modified the validity estimates.

**Methods:**

The study comprised a total of 302 adolescents from an urban and rural area in Ecuador. Validity was evaluated by comparing the record with accelerometer recordings for seven consecutive days. Test-retest reliability was examined by comparing registrations from two records administered three weeks apart. Time spent on sedentary (SED), low (LPA), moderate (MPA) and vigorous (VPA) intensity physical activity was estimated. Bland Altman plots were used to evaluate measurement agreement. We assessed if age, sex, urban or rural setting, anthropometry and convenience of completing the record explained differences in validity estimates using a linear mixed model.

**Results:**

Although the record provided higher estimates for SED and VPA and lower estimates for LPA and MPA compared to the accelerometer, it showed an overall fair measurement agreement for validity. There was modest reliability for assessing physical activity in each intensity level. Validity was associated with adolescents’ personal characteristics: sex (SED: *P* = 0.007; LPA: *P* = 0.001; VPA: *P* = 0.009) and setting (LPA: *P* = 0.000; MPA: *P* = 0.047). Reliability was associated with the convenience of completing the physical activity record for LPA (low convenience: *P* = 0.014; high convenience: *P* = 0.045).

**Conclusions:**

The physical activity record provided acceptable estimates for reliability and validity on a group level. Sex and setting were associated with validity estimates, whereas convenience to fill out the record was associated with better reliability estimates for LPA. This tendency of improved reliability estimates for adolescents reporting higher convenience merits further consideration.

## Background

The benefits of physical activity (PA) on health and its role in disease prevention are widely acknowledged [1,2]. Adequate levels of PA are associated with a reduced risk of chronic diseases and all-cause mortality [1,3,4]. An estimated three million deaths each year could be prevented if people were sufficiently active [5]. In particular, regular PA at a young age can prevent chronic diseases and improve mental well-being during childhood and later in life [6,7]. However, recent data on self-reported PA suggests that only 30% to 40% of young people are sufficiently active worldwide [8]. In young people in low- and middle-income countries (LMICs) physical inactivity rates are high, particularly among girls in countries in Latin-America [1,9], and already constitute one of the leading causes for morbidity [9,10] and premature death [11].

To determine the risk for chronic diseases and effectiveness of preventive interventions, a valid and feasible assessment of PA is crucial [12]. However, PA assessment in young people is challenging, regardless of whether objective or subjective measures of PA are used [8,13]. Accelerometer registrations are now widely implemented for objective assessment of PA in children and are recognized as an appropriate measure for PA surveillance on a population level [12]. Nonetheless, to estimate PA behavior in large epidemiological studies, self-reported measures remain common. Before such subjective instruments can be applied, they should first be validated against an objective criterion method such as accelerometers. The majority of validation studies in LMICs were performed in adults and report low to moderate measurement agreement for questionnaires [14-16]. The very few validation studies in young people using questionnaires and other self-reported instruments have shown poor to moderate validity [17-19]. However, none of these studies explored or identified possible reasons for this low validity thereby hampering a better understanding of PA assessment and validity of the instruments used in these settings.

The present study was carried out in the context of ACTIVITAL!, a pair-matched, cluster-randomized controlled trial in school-going adolescents in Ecuador. This intervention aims to promote health, particularly by improving diet and PA. As the socio-cultural and physical environment in Ecuador is distinct from high-income countries and adopting existing tools cannot guarantee validity of the proposed measures in this population [20], a validation study of a PA record (also called diary) was conducted. The accelerometer was chosen as an objective instrument to validate the record. The choice for using both these instruments was based on the existing evidence, the age category under investigation, the study design, resources and staff available [21]. The aim of the present study was to (i) assess the validity and reliability of a PA record as an instrument to estimate PA on a group level in an Ecuadorian adolescent population, and (ii) explore which factors are associated with this validity and reliability.

## Methods

The recently published Hagströmer-Bowles Physical Activity/Sedentary Behavior Questionnaire Checklist [22] was followed to report this study. It provides key methodological quality criteria for validation studies of instruments examining self-reported PA and/or sedentary behavior.

### Participants

**Figure 1:**
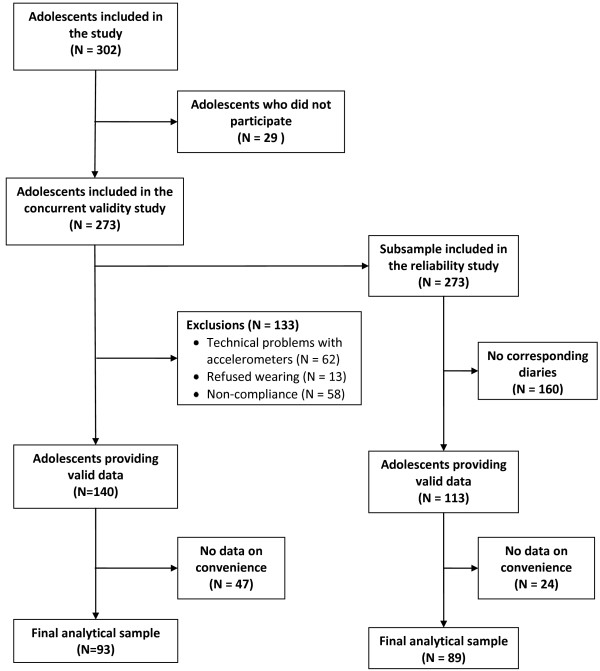
Flow chart of the study design.

A convenience sample of 302 school-going adolescents, aged 11–15 years, was recruited from a rural (Nabón; *N* = 70) and an urban (Cuenca; *N* = 232) area in the Azuay province of the Ecuadorian Andes region (Figure 1). Seven mixed gender schools, four urban and three rural, were selected for the study. Except for one private urban school, all were public schools. All children aged 11–15 years (from grade 7 to 11) in the schools were invited to participate. The exclusion criteria used were: (i) having a medical condition that hampered physical activity or (ii) being pregnant. Parental informed consent forms were distributed to the children that were eligible for the study. Those who returned signed parental consent forms (*N* = 302) and completed individual assent forms (*N* = 302) were included in the study. The study took place from April to July in 2008 and was approved by the Ethics Committee of the Ghent University Hospital (B67020084010).

### Design and procedures

The overall validity of the PA record was assessed by (i) comparing it with the accelerometer recordings (validity) and (ii) comparing the two administrations of the record (test-retest reliability). In addition, we used a socio-demographic and Likert scale questionnaire to explore whether overall validity was affected by factors at individual (age, sex, Body Mass Index (BMI) and perceived convenience to complete the PA record) and environmental (setting) level.

Data collection was organized during school hours on three occasions, i.e. (i) on the first day of the study, (ii) after one week of accelerometer measurement, and (iii) after three weeks. On the first day, we provided classroom demonstrations and instructions on how to wear the accelerometer and complete the PA record to both the participants and their teachers. All participants were instructed to complete the PA record for seven consecutive days and wear the accelerometer during the same time period, i.e. both instruments were temporally matched. During the measurement period, teachers and researchers regularly reminded the participants in class of the importance of completing the record as soon as possible after activities had ended. In addition, socio-demographic data (age, sex and setting) and anthropometry were collected for each participant on the first day. On the second visit, i.e. after one week, both accelerometers and the completed PA record were collected from the participants. The data from those students who (i) were absent, (ii) were not wearing the accelerometer or (iii) did not have their PA record with them on this day, were collected during additional visit(s) to the schools. Finally, three weeks after the first visit, the PA record was administered a second time to assess reliability. To maximize comparability of both PA record administrations, the same procedures were applied (i.e. use of the same measuring instrument, the same researcher explaining the PA record) under the same conditions at school (e.g. no holidays or special activities). During this second administration, a Likert scale questionnaire was administered to examine perceived convenience as a predictor of validity and reliability.

### Anthropometric measurements

Anthropometric measurements were carried out in duplicate by two independently trained researchers while ensuring optimal privacy. Adolescents wore light clothing but no shoes during the measurements. Body height was measured and recorded to the nearest 0.1 cm with a portable stadiometer and body weight to the nearest 0.1 kg using a digital calibrated balance (model SECA 803, Seca GmbH & CO, Hamburg, Germany). Adolescents were classified, based on age and sex, into BMI categories for 11–15 year old adolescents (underweight, normal weight, overweight and obese) [23,24].

### PA record

To assess PA levels, a simplified version of a previously validated PA record was used [25]. The list of pre-defined common activities and related numerical codes present in the original PA record were omitted in our study. On the form, each day was divided into 96 intervals of 15 min with space available to report performed activities. The instrument and related instructions were provided in Spanish. To limit recall bias, participants were instructed to record all types of activities performed during 24 hours for 7 consecutive days either directly after finalizing the activities or as soon as possible after the activities had ended.

For the analysis, each 15 min activity interval of the PA record was converted to a MET-value using the compendium of energy expenditure values for young people [26], and classified into durations by multiplying the estimated MET value of the activity by the time engaged in it. Due to logistical constraints at the schools, the first and the last measurement day started and ended at 12:00 noon and were therefore omitted from analysis. To ensure comparability of total estimated time of the record and the accelerometer registrations, only the daily active time reported by the record was included. In addition, all days with less than nine hours (540 min) were omitted from analysis. Time (MET-min/day) spent on sedentary PA (SED) (≤ 1.5 METs), low intensity PA (LPA) (≥1.5 and < 3 METs), moderate intensity PA (MPA) (≥ 3 and < 6 METs) and vigorous intensity PA (VPA) (≥ 6 METs) were computed as outcome variables [27,28].

### Accelerometers

An objective assessment of PA levels was obtained using the uniaxial GT-256 and GT1M ActiGraph accelerometers (Actigraph Manufacturing Technology Incorporated, Fort Walton Beach FL, USA). These accelerometers are appropriate to measure PA behavior in adolescents and are considered comparable for the evaluation of intensity levels [29]. On the first day of the study and after measuring anthropometry, pre-initialized accelerometers were distributed and placed on the right side of the hip using an adjustable elastic belt. Participants received a demonstration from a trained researcher on how to wear the accelerometer. Instructions provided were for example “accelerometers could only be removed when sleeping, showering or engaging in other water activities”, “do not clean the accelerometer with a solvent”, “always wear the accelerometer on the same place on your waist”. In accordance with the protocol of the PA record, the accelerometers were programmed to initialize at 12:00 noon on the first measurement day and were set to register 1-minute epoch cycles.

### Possible predictors of validity and reliability estimates

A Likert scale questionnaire was developed based on two focus groups (results not included in this study). Focus groups were conducted with participants that were different from those who participated in this study. These focus groups explored the factors that might compromise or promote the validity of any self-reported PA assessment. The focus groups allowed us to use appropriate language and tailor the questionnaire to this specific age group. This final questionnaire assessed the degree of difficulty (i.e. convenience) for the adolescents to complete the record by including questions on: (i) their time perception, (ii) recall bias and (iii) social norms/desirability. We used the following Likert scale response categories: 1 “strongly disagree”, 2 “disagree”, 3 “neutral”, 4 “agree”, 5 “strongly agree” and 6 “I don’t know”. A convenience scale (Cronbach’s α = 0.59) combining these questions was used to provide a comprehensive assessment of the degree of difficulty of completing the PA record. Children with a higher score on this scale were those who provided answers reflecting the most favorable conditions for completing the PA record. To explore the association of convenience with reliability and validity, tertiles from this scale were created. Finally, tertiles of participants who found it respectively the least, more or less, and the most convenient to complete the PA record were compared with one another.

### Accelerometer data reduction

The Actilife Software (Actigraph Manufacturing Technology Incorporated, Fort Walton Beach FL, USA) was used to process the accelerometer data and computed both total registered time and time spent in different intensity levels of PA. Non-wearing time for the accelerometers was defined as 60 min of continuous zero values, allowing for 1–2 min registrations of less than 10 counts [30]. Accelerometers were considered malfunctioning if no counts were registered or when a constant number of counts were recorded during the whole day (*N* = 62) (Figure 1). As with the PA record, both the first and the last day of the administration period were excluded and all days with less than nine hours (540 min) were omitted from analysis (*N* = 2). The following cut-off points were adopted from other studies in this age-group to determine the time spent on different intensity levels of PA: SED (≤ 100 counts) [31,32], LPA (101–759 counts) [32], MPA (760–4011 counts) [32,33] and VPA (≥ 4012 counts) [33]. The cut-off point for MPA was chosen to detect moderate intensity non-ambulatory and ambulatory activities with high sensitivity and specificity [32]. The cut-off point for VPA was chosen to detect ambulatory vigorous intensity activity with high specificity for the age-group [33].

### Data analysis

Data from the PA record were entered in double using Epi data (Version 3.14, Odense Denmark) and analyzed using Stata (Intercooled Stata version 12 Statacorp, college station, TX, USA). PA registrations were included if subjects had at least two days of corresponding accelerometer and PA record recordings. This inclusion criterion was chosen as the study aimed to estimate PA on a population level with as many subjects as possible. In this case, our criterion is acceptable even though this means having fewer valid days and higher individual variance [21]. As measurement agreement might vary with the total time captured, the measured time for each intensity level was standardized to 900 min, which corresponds to the assumption of nine hours of sleep a day. All analyses were performed using these standardized outcome measures for both the accelerometers and PA record. Descriptive data were reported as mean and SD. We tested differences in means between methods (validity) and repeated measures (reliability) using linear mixed effects models with the levels *school* and *individual*. Standard errors were estimated using the Huber-White sandwich estimator that relaxes distributional assumptions of homoscedasticity of the model residuals [34]. Statistical significance was set at an alpha level of 0.05 and all tests were two-sided.

The measurement agreement between the duration of each PA intensity level was examined for both validity and reliability using the Bland Altman diagnostic plots. The plots visualize the difference between the PA measurements (validity: PA record 1 – accelerometer; reliability: PA record 1 – PA record 2) against their average values. In case plots showed a tendency for the differences to increase as the magnitude of measurement increased, data were log-transformed and re-plotted [35]. In the latter case, the mean difference and Limits Of Agreement (LOA) were back transformed by taking the antilog and values were presented as percentages. In case of a linear trend between the differences and the mean of two measurements, the differences were regressed over the means to obtain LOA that are a linear combination of the mean of the two measurements (A) [35]. Lowess curves were used to visualize any group differences in validity and reliability. Classification agreement for both validity and reliability was further examined using linear weighted Kappa statistics and its 95% CI, based on groups defined by tertiles of SED, LPA, MPA and VPA. Strength of agreement for the kappa coefficient was evaluated using the standards as proposed by Landis and Koch [36]. To account for prevalence and bias effects the prevalence-adjusted and bias-adjusted kappa (PABAK) is presented alongside the kappa statistics [37]. Finally, we assessed the association between gender, convenience level (in tertiles), age, setting (urban vs. rural) and BMI with reliability and validity. For this purpose we used the differences (validity: PA record 1 – accelerometer; reliability: PA record 1 – PA record 2) for each PA intensity level between 2 measurements as an outcome variable. These analyses were performed separately for each intensity level using linear mixed effects models with robust estimation for SE to account for clustering of estimates at the school level [34].

## Results

### Characteristics of participants

Figure 1 visualizes the number of adolescents included and analyzed in the study. After data reduction, a total of 140 adolescents (52.1% male) provided valid data for the first administration of the PA record and the accelerometer (Table 1). The sample included 101 adolescents (72%) from an urban and 39 from a rural area (28%). Mean age of the participants was 13.4 ± 1.3 years and mean BMI was 20.6 ± 3.7 kg/m^2^. On average, 6.7%, 13.4% and 1.5% of the adolescents were obese, overweight and underweight respectively.

**Table 1:** Participant characteristics

	***N***	**Total**		***N***	**Urban**		***N***	**Rural**		*P*-value*
		**Mean**	**SD**		**Mean**	**SD**		**Mean**	**SD**	
Age (year)	140	13.4	1.3	101	13.3	1.2	39	13.6	1.6	0.760
Weight (kg)	140	46.0	11.9	101	47.6	12.2	39	41.9	10.1	0.216
Height (cm)	134	148.8	9.1	95	150.3	8.6	39	145.1	9.2	0.217
BMI (kg/m^2^)	134	20.6	3.7	95	20.9	4.0	39	19.6	2.8	0.251

*P-values for urban–rural differences.

A subsample of 113 adolescents (48.7% male) provided data for both PA records (Figure 1). There was no difference between the adolescents that provided data for both PA records and those who had data on the first PA record and accelerometer in terms of mean age (*P* = 0.54), weight (*P* = 0.68), height (*P* = 0.22) and BMI (*P* = 0.97). Finally, there was no significant difference in the convenience score between those participants included in the final sample and those participants initially recruited (*P* = 0.84).

### Descriptive PA estimates

On average, 871 min (14 h 31 min) were captured by the first PA record, and 792 min (13 h 12 min) by the accelerometer, indicating a higher mean total reported time for the PA record than for the accelerometer. For those providing repeated measures of the PA record (*N* = 113), the first PA record (866 min) provided lower total time estimates compared to the second (892 min) PA record. The PA estimates after standardization are shown in Table 2. On average, half of the reported time measured by the first PA record and the accelerometer was spent as SED. The PA record gave significantly higher estimates than the accelerometer for SED and VPA, and significant lower estimates for LPA. Only for MPA similar estimates were provided by both instruments. When looking at repeatability, the first PA record reported less SED and consequently more LPA, MPA and VPA compared to the second record. However, only a significant difference was found for SED and LPA (Table 2).

**Table 2:** Average standardized time (minutes per day) of activity reported for validity and reliability

**PA intensity (min/day)**	**Validity**					**Reliability**				
	**PA record 1 (*N*** **= 140)**		**Accelerometer (*N*** **= 140)**		***P*-value**	**PA record 1 (*N*** **= 113)**		**PA record 2 (*N*** **= 113)**		***P*-value**
	**Mean**	**SD**	**Mean**	**SD**		**Mean**	**SD**	**Mean**	**SD**	
SED	488	109	432	90	0.001	482	120	550	136	0.002
LPA	192	88	265	48	0.011	201	99	158	83	0.023
MPA	175	104	188	64	0.351	170	104	155	111	0.501
VPA	45	69	15	16	0.003	46	65	37	57	0.127

PA: Physical Activity; SED: Sedentary Intensity Physical Activity; LPA: Low Intensity Physical Activity; MPA: Moderate Intensity Physical Activity; VPA: Vigorous Intensity Physical activity.

### Validity

**Figure 2:**
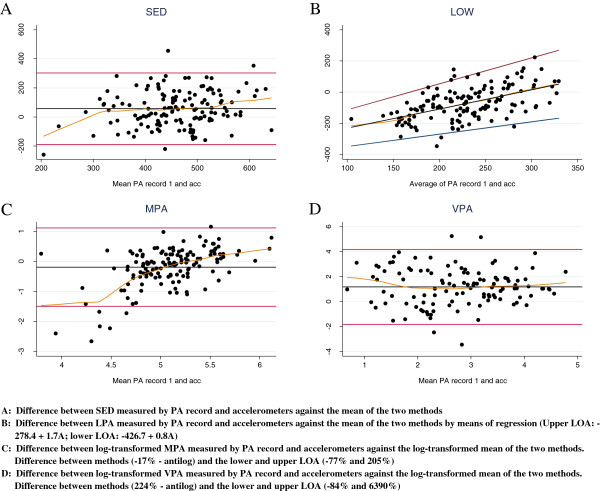
**Validity and convenience of the PA record versus accelerometer for adolescents (*N*** **= 140). PA: Physical Activity; LOA: 95% Limits of Agreement; SED: sedentary Intensity Physical Activity; LPA: Low Intensity Physical Activity; MPA: Moderate Intensity Physical Activity; VPA: Vigorous Intensity Physical Activity.**

Results for validity are provided for those participants with data on both the PA record and accelerometers (*N* = 140) (Figure 2). On average, the PA record estimated 57 min (95% CI: [36;77] and LOA [-189;303]) more time spent on SED. For LPA, differences between both methods increased when more LPA was reported (β = 1.22; CI [0.91; 1.54]; lower LOA: -426.7 + 0.8A; upper LOA: -278.4 + 1.7A). Log transforming MPA showed that record estimates were on average 17% (CI [-26%; -8%]) lower than the accelerometers, but LOA were wide [-77.5%; 205%]. For lower mean MPA, adolescents tend to under report whilst for higher mean MPA they over report. For VPA the measurement disagreement between the PA record and accelerometer increased with increasing time measured. After log transforming, it is clear that the record reported more time spent on VPA (224%; CI [145%; 327%]) compared to the accelerometer. For VPA, the wide LOA [-84%; 6390%] indicated large discrepancies between both methods for some individuals.

Kappa statistics analyzing the classification agreement between the accelerometer and the first PA record showed fair to moderate agreement. The kappa coefficient improved for all categories when adjusted for prevalence and bias (Table 3).

**Table 3:** Cohen’s kappa, 95% CI and prevalence-adjusted and bias-adjusted Cohen’s kappa (PABAK) for each activity category

	**Cohen’s Kappa**	**95% CI**	**PABAK**
**Validity (accelerometer vs. PA record 1)**			
SED	0.45	0.33 – 0.57	0.52
LPA	0.36	0.25 – 0.48	0.44
MPA	0.46	0.34 – 0.58	0.53
VPA	0.45	0.33 - 0.57	0.52
**Reliability (PA record 1 vs. PA record 2)**			
SED	0.58	0.44 – 0.72	0.63
LPA	0.61	0.47 – 0.75	0.66
MPA	0.49	0.35 – 0.63	0.55
VPA	0.36	0.21 -0.48	0.50

SED: Sedentary Intensity Physical Activity; LPA: Low Intensity Physical Activity; MPA: Moderate Intensity Physical Activity; VPA: Vigorous Intensity Physical activity.

### Reliability

**Figure 3:**
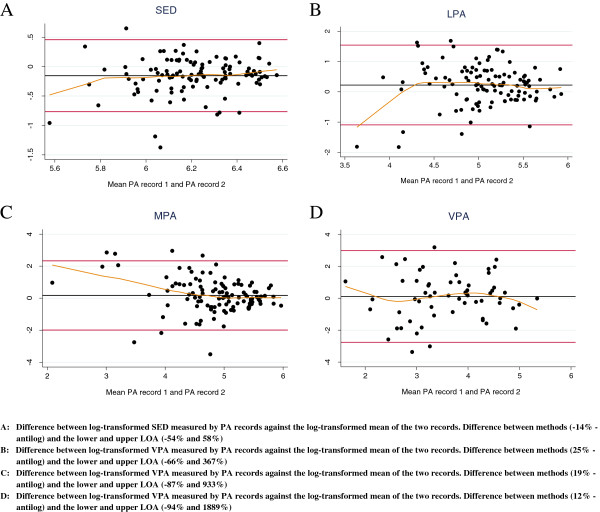
**Reliability and convenience of the PA record for adolescents (*N*** **= 113) PA: Physical Activity; LOA: 95% Limits of Agreement; SED: sedentary Intensity Physical Activity; LPA: Low Intensity Physical Activity; MPA: Moderate Intensity Physical Activity; VPA: Vigorous Intensity Physical Activity.**

Results for reliability are provided for those participants with data on both PA records (*N* = 113) (Figure 3). After log transformation, the first PA record reported significantly less time spent on SED (mean difference -14%, CI: [-19%, -9%] and LOA [-53%; 58%]) than the second PA record. The SED plot showed fair agreement as both mean difference and LOA were within acceptable limits. For LPA the first record significantly exceeded the second with 25% (CI: [11%, 42%]) with LOA [-66%; 367%]. The plots of MPA and VPA also showed how the mean bias was acceptable for the different intensities of PA. For MPA and VPA the mean differences of the first PA record were respectively 19% (CI: [-3%; 46%] and 12% (CI: [-23%; -52%]) higher than the registrations of the second PA record. Even though these mean differences were acceptable, the LOA for MPA [-87%; 933%] and VPA [-94%; 1889%] indicated large discrepancies between both records in individuals. The differences between the repeated measures decreased with higher mean estimates of MPA.

When comparing both PA records, an overall moderate classification agreement was found. The kappa statistics improved when taking into account the impact of prevalence and bias in determining the magnitude of the kappa coefficient (Table 3).

### Predictors of validity

For validity (Table 4), sex was significantly associated with SED, LPA and VPA. Girls reported more LPA time (73 min) and less SED time (73 min) and VPA time (33 min) than boys. Setting was significantly associated with LPA and MPA. Rural participants reported on average more LPA time (100 min) and less VPA time (52 min) than their urban peers. Adolescents who reported higher convenience to complete the PA record did not produce a different validity compared to peers reporting lower convenience. Importantly, those adolescents with the highest convenience level complied better with the protocol for wearing the accelerometers and had more registered days.

**Table 4:** Predictors of measurement agreement for standardized PA record and accelerometer recordings

**Predictors**	**Full model PA record 1 - accelerometer**^**a**^							
	**SED**		**LPA**		**MPA**		**VPA**	
	**β**	***P*-value**	**β**	***P*-value**	**β**	***P*-value**	**β**	***P*-value**
**Female**	-72.6	0.007	73.4	0.001	32.6	0.146	-33.4	0.009
**Age**	-6.1	0.590	2.6	0.782	5.5	0.553	-2.1	0.689
**Rural**	-49.6	0.115	99.9	<0.001	-51.8	0.047	1.6	0.916
**BMI**	-6.3	0.094	3.4	0.285	2.1	0.492	0.7	0.688
**Convenience_1**^**b**^	-30.4	0.374	27.4	0.347	-6.1	0.829	9.1	0.574
**Convenience_2**^**b**^	3.2	0.918	14.8	0.575	-37.9	0.139	20.1	0.171

^a^Linear mixed model with school as random effect.

^b^Convenience categories using the lowest convenience category as reference.

SED: Sedentary Intensity Physical Activity; LPA: Low Intensity Physical Activity; MPA: Moderate Intensity Physical Activity; VPA: Vigorous Intensity Physical activity.

### Predictors of reliability

Reliability was not associated with adolescents’ individual characteristics such as age, sex, and BMI, and environmental characteristics, such as setting (Table 5). However, the measurement agreement improved significantly for adolescents who reported a higher convenience of completing the record compared to their peers who reported more difficulties for MPA. Compliance with the PA protocol did not differ between the different convenience levels. There was no difference in number of registered days between the convenience groups.

**Table 5:** Predictors of measurement agreement for both standardized PA records

**Predictors**	**Full model PA record 1 – PA record 2**^**a**^							
	**SED**		**LPA**		**MPA**		**VPA**	
	**β**	***P*-value**	**β**	***P*-value**	**β**	***P*-value**	**β**	***P*-value**
**Female**	-19.8	0.521	22.9	0.317	0.6	0.982	-5.4	0.701
**Age**	-7.7	0.612	-3.8	0.766	8.0	0.596	10.2	0.142
**Rural**	28.0	0.441	23.9	0.595	-58.0	0.356	-19.3	0.246
**BMI**	-3	0.489	2.11	0.513	1.5	0.673	-1.0	0.615
**Convenience_1**^**b**^	34.9	0.377	-73.3	0.014	12.3	0.715	26.2	0.147
**Convenience_2**^**b**^	82.5	0.027	-57.3	0.045	-40.1	0.216	16.8	0.322

^**a**^Linear mixed model with school as random effect.

^b^Convenience categories using the lowest convenience category as reference.

SED: Sedentary Intensity Physical Activity; LPA: Low Intensity Physical Activity; MPA: Moderate Intensity Physical Activity; VPA: Vigorous Intensity Physical activity.

## Discussion

We evaluated the validity and reliability of a PA record for its application to assess PA at a group level in Ecuadorian adolescents and examined factors at individual and environmental level that might modify this. Our results showed that measurement agreement for validity and reliability was satisfactory at a group level, and classification agreement was fair to moderate. There was no trend of over or under reporting for repeatability and validity at the different intensity levels, except for MPA. Participants’ age, BMI and convenience to fill out the form did not modify validity. However, sex (for SED, LPA and VPA) and setting (for LPA and MPA) were associated with validity. In addition, we found that only convenience was associated with higher reliability for LPA while sex, setting, BMI and age had no influence on the reliability estimates.

On average, the record measurements were substantially higher for SED and VPA compared to the accelerometer’s measurements, whilst these were lower for LPA and MPA. The differences were significant except for MPA. These observations are consistent with previous findings that LPA or MPA were underestimated during self reporting. VPA was consistently overestimated, suggesting a misclassification of MPA as VPA [13,38]. In our study, LPA was underestimated and its mean difference surprisingly increased with higher reported LPA, as observed by Krishnaveni *et al.*[17]. Furthermore, the underestimation of MPA by the record is possibly explained by the fact that the time spent at lower intensity levels is not as easily remembered, quantified and subsequently accurately reported as structured PA, like exercise and sports. LPA and MPA are thus less likely to be included when using self-reported measures [13]. Finally, our results for VPA showed a difference between both methods, which increased with higher mean time reported. Anderson *et al.*[39] identified several factors that could contribute to such higher estimates of VPA. Firstly, children generally do not engage in sustained VPA. Their PA pattern is characterized by very short outbursts of intense PA alternated with varying intervals of LPA and MPA [40]. Children might intuitively, but wrongly, acknowledge the majority of this period as VPA, which likely influences record estimates. Secondly, accelerometers have limited ability to detect some vigorous activities such as swimming, cycling, and movements of the torso or locomotion on a gradient [41]. Particularly walking uphill or carrying heavy loads are examples of such activities performed by the study population which could have been underestimated by the accelerometer. In addition, children could have removed the accelerometers during vigorous activities out of fear of damaging the device.

Next to our validity, the observed reliabilities were satisfactory except for MPA. A previous study reporting reliability using the Bland Altman method showed fair agreement for all intensity levels [19]. The wide LOA and their tendency to increase with higher PA intensity however, indicate that the validity of our PA record is limited for individual observations, in particular those at higher intensity. Despite these large differences in individual observations, we consider the measurement agreement for validity and reliability satisfactory at a group level.

We expected that personal factors at individual (i.e. age, sex, BMI and self-reported convenience) and environmental level (i.e. setting) could alter both reliability and validity. For validity our hypothesis was not confirmed statistically for convenience, age and BMI, but it was significantly different for sex and setting at specific intensity levels. Our findings did not show any age effect on validity, which is in contrast to a previous study where younger adolescents had a larger median difference in total time spent in PA than their older peers [42]. A previous study examining the associations of validity between a self-reported 7-day physical activity questionnaire and accelerometers in children showed that sex and body fat did not affect the validity estimates [43]. While the former study reported no effect of sex on validity, a systematic review on PA measures in children showed that female participants were likely to overestimate their activity [38]. Our results indicated that female participants over reported LPA, but under reported SED and VPA. Poor validity in rural areas, as observed in our study for LPA and VPA, has been reported in a study evaluating validity of a questionnaire in Vietnamese adolescents [18]. We also note that only for LPA the highest convenience group had better reliability estimates compared to the other groups. A previous study that investigated self-reported confidence in recalling PA as a predictor of validity showed that participants in the high-confidence group had higher validity and repeatability coefficients than those in the low-confidence group for most comparisons [44].

The current study has a number of strengths. First, the PA record was validated against an objective measure of PA. Furthermore, we used cut-off points for MPA that include both ambulatory and non-ambulatory moderate intensity activities [32]. Other studies generally used cut-off points based upon walking and running at different intensities for MPA [13]. Second, PA records or activity diaries were previously reported to estimate PA accurately at population level in adolescents [39]. However, using this type of self-reported measure does not come without disadvantages. It imposes a higher participant burden, which might in turn affect their behavior (Hawthorne effect). Third, as mentioned previously, the frequent activities of short duration might provide lower estimates then accelerometers, as only the major activity of each 15 min time interval will be reported [45]. However, introducing even shorter time-intervals would render completing the PA record even more burdensome. Lastly, we did not present correlation coefficients in this study. Not doing so may limit comparability with other similar studies. We believe however that this is irrelevant as they are inappropriate to assess measurement agreement as they only measure the strength of linear association between variables [46].

## Conclusion

The physical activity record provided acceptable estimates on a group level. Sex and setting were the characteristics associated with differences in validity for SED, LPA, and VPA and LPA and MPA, respectively. Convenience was associated with lower differences in reliability. However, the interesting finding of better validity for the highest convenience group when reporting LPA merits further exploration. Adequately powered longitudinal studies combined with direct observation examining convenience are needed. As such, new insights into poor validity estimates might be achieved and would contribute to a better understanding of physical activity assessment and validity of the instruments used in LMIC.

## Abbreviations

PA: Physical activity; BMI: Body mass index; LMICs: Low- and middle-income countries; MET: Metabolic equivalent; SED: Sedentary intensity physical activity; LPA: Low intensity physical activity; MPA: Moderate intensity physical activity; VPA: Vigorous intensity physical activity; CI: Confidence interval; LOA: Limits of agreement; PABAK: Prevalence-adjusted and bias-adjusted kappa.

## Competing interests

The authors declare that they have no conflict of interest. RV, AO and SA have received a grant from VLIR-IUC. SD has received an honoraria from VLIR-IUC. The research was funded by VLIR-IUC and Nutrition Third World. The funding source was not involved in the design, collection, analysis, interpretation of the data or the writing of the manuscript.

## Authors’ contributions

RV, CL, OA and PK were responsible for the design of the study, the development of the PA record and the methodology. RV, CL, LH and MH carried out the statistical analysis. OA participated in the design and coordination of the study and helped to draft the manuscript. LM, SD, PK and SA assisted in the interpretation of the data. RV drafted the initial paper and has the responsibility for the final content. All authors contributed to the interpretation of the results and critically reviewed and approved the final manuscript.

## Pre-publication history

The pre-publication history for this paper can be accessed here:

http://www.biomedcentral.com/1471-2458/13/1109/prepub
